# Structure and Content of Drug Monitoring Advices Included in Discharge Letters at Interfaces of Care: Exploratory Analysis Preceding Database Development

**DOI:** 10.2196/10832

**Published:** 2019-04-08

**Authors:** Benedict Morath, Katharina Wien, Torsten Hoppe-Tichy, Walter Emil Haefeli, Hanna Marita Seidling

**Affiliations:** 1 Department of Clinical Pharmacology and Pharmacoepidemiology Heidelberg University Hospital Heidelberg University Heidelberg Germany; 2 Cooperation Unit Clinical Pharmacy Heidelberg University Hospital Heidelberg University Heidelberg Germany; 3 Hospital Pharmacy Heidelberg University Hospital Heidelberg University Heidelberg Germany

**Keywords:** drug monitoring, patient discharge summaries, transition of care

## Abstract

**Background:**

Inadequate drug monitoring of drug therapy after hospital discharge facilitates adverse drug events and preventable hospital readmissions.

**Objective:**

This study aimed to analyze the structure and content of drug monitoring advices of a representative sample of discharge letters as a basis for future electronic information systems.

**Methods:**

On 2 days in November 2016, all discharge letters of 3 departments of a university hospital were extracted from the hospital information system. The frequency, content, and structure of drug monitoring advices in discharge letters were investigated and compared with the theoretical monitoring requirements expressed in the corresponding summaries of product characteristics (SmPC). The quality of the drug monitoring advices in the discharge letters was rated with the domains of an adapted systematic instructions for monitoring (SIM) score.

**Results:**

In total, 154 discharge letters were analyzed containing 1180 brands (240 active pharmaceutical substances), of which 50.42% (595/1180) could theoretically be amended with a monitoring advice according to the SmPC. In reality, 40 discharge letters (26.0%, 40/154) contained a total of 66 monitoring advices for 57 brands (4.83%, 57/1180), comprising 18 different monitoring parameters. Drug monitoring advices only addressed mean 1.9 (SD 0.8) of the 7 domains of the SIM score and frequently did not address reasons for monitoring (86%, 57/66), the timing of monitoring, that is, the start (76%, 50/66), the frequency (94%, 63/66), the stop (95%, 63/66), and how to react (83%, 55/66).

**Conclusions:**

Drug monitoring advices were mostly absent in discharge letters and a gold standard for appropriate drug monitoring advices was lacking. Hence, more effort should be put in the development of tools that facilitate easy presentation of clinically meaningful drug monitoring advices at the point of care.

## Introduction

### Background

Adverse drug events (ADE) frequently occur after the patient transitions across interfaces of care, thus making patients prone to unintended outcomes such as hospital readmissions [1,2]. Indeed, up to 10% of all hospital readmissions occur as a consequence of ADE, and nearly 1 in 4 of these ADE is caused by drugs just started during the index hospitalization [1,3-5]. Since during hospitalization, more than 95% of all prehospital drug therapies are modified, appropriate follow-up monitoring is particularly important [6-9]. Furthermore, in the discharge medication, over half of the drugs are newly prescribed during hospitalization, emphasizing the need for closer monitoring during the initial postdischarge phase [8]. However, after hospital discharge, monitoring of safety (ADE) and efficacy is often lacking, thus causing potentially preventable readmissions [10]. Interestingly, in the ambulatory setting, preventable ADE resulting from inadequate monitoring and leading to hospitalization are more likely associated with commonly prescribed drugs such as drugs with a cardiovascular indication [1,3,11-13]. For instance, about one-third of patients treated with angiotensin-converting-enzyme (ACE) inhibitors do not undergo serum creatinine and potassium controls at least yearly; although, it is well established that monitored patients experience ADE less often [14-17].

### Objectives

Hence, it appears useful to include structured and comprehensive drug monitoring advices in discharge letters concerning the safety and efficacy of drug therapy to support general practitioners with drug therapy monitoring and ensure a safe patient transfer across the interfaces of care. Today, the current state of drug monitoring recommendations at interfaces is not well known and except for specific diseases or drugs, a comprehensive and prospectively evaluated gold standard for evidence-based drug monitoring advices is lacking [18,19]. As a first step to develop and subsequently provide suitable drug monitoring advices at interfaces of care, we performed an exploratory analysis of the structure and the patterns of current drug monitoring advices in discharge letters and compared this information with the statutory information provided in the pertinent summary of product characteristics (SmPC).

## Methods

### Context

We analyzed an exploratory sample of consecutive discharge letters of 3 major departments of a large university hospital to determine the number, structure, and content of the drug monitoring advices that are currently provided in daily practice. Therefore, discharge letters of the divisions of hemato-oncology, gastroenterology, cardiology, endocrinology, general medicine, psychosomatics, visceral surgery, vascular surgery, cardiac surgery, urology, and neurology were included in the analysis. Although a German drug monitoring guideline was published in 2013 by the German College of General Practitioners and Family Physicians, a comprehensive and prospectively evaluated gold standard for evidence-based drug monitoring advices in different settings of care is not established at present in Germany [20]. Therefore, the information in discharge letters was compared with the generic drug monitoring parameters of the SmPC. This study was approved by the responsible Ethics Committee of the Medical Faculty of Heidelberg University (S-402/2016).

### Setting and Data Collection of Drug Monitoring Parameters in the Discharge Letter

As a point prevalence analysis, all final discharge letters of the departments of, surgery, internal medicine, and neurology that were issued on November 15 and November 16 of the year 2016 and stored in the hospital information system were screened by 1 author. The departments were chosen to cover a broad spectrum of medications of different specialties and generate a representative overview. All discharge letters containing a discharge medication were selected, printed, and pseudonymized by blacking data of the attending physicians and the patient and attributing a consecutive number code to every letter.

The entire discharge letter was independently read by 2 investigators and screened for drug monitoring advices. The following information was extracted into a predefined Microsoft Excel (Microsoft Corporation) sheet with the following categories: Code of the discharge letter, name of all drugs listed as discharge medication including their strength, dosage, and additional information such as administration advices, as well as potential drug monitoring advices with their content and placement in the letter (eg, directly adjunct to a drug or included in the prose text).

A drug monitoring advice was defined as a statement that was explicitly (eg, “please monitor serum potassium under ramipril therapy”) or by placement (ie, proximity) connected with the recommended drug treatment at discharge. Second, a drug monitoring advice needed to explicitly state tests that should be performed (eg, electrocardiogram) or parameters that should be checked (eg, potassium) either in terms of safety and ADE monitoring (eg, liver function test to detect hepatotoxicity) or in terms of efficacy (eg, target low-density lipoprotein values to identify poor or nonresponders).

We did not differentiate in drug monitoring advices for newly prescribed drugs and those that were already on the patients’ medication list at the time of hospital admission.

### Structure and Content of Drug Monitoring Advices in Discharge Letters

To determine the structure and content of current drug monitoring advices, the drug monitoring advices were independently categorized by 2 authors using the domains of an adapted version of the systematic instructions for monitoring (SIM) score [21]. The SIM score contains 7 essential domains of information, which should be addressed in an unequivocal and comprehensive drug monitoring advice: (1) why to monitor, (2) what to monitor, (3) when to start monitoring, (4) how frequently to monitor, (5) what to look for, that is, target values in terms of drug efficacy or specific ADE such as laboratory changes, (6) how to respond to findings, and (7) when to stop drug monitoring. When analyzing the drug monitoring advices, we specified for every category whether it was included in the drug monitoring advice (=1 point) or not (=0 points).

### Extraction of Summary of Product Characteristics Information

The SmPC of all brands reported in the discharge medications were independently screened by 2 authors. When no brand name and only an active pharmaceutical substance was provided in the discharge medication, the SmPC of the brand listed in the hospital formulary was screened because this was the last specific brand the patient received. All eligible text passages concerning drug monitoring of the respective brands were transferred into an excel sheet once a consensus of the 2 reviewers was reached. If consensus was not reached, a third reviewer was involved.

In analogy to the discharge letters, a drug monitoring advice was defined as a parameter that should be measured or a test that should invariably be performed for safety or efficacy reasons at a given time during or after the treatment. Extracted drug monitoring parameters and tests are available in Multimedia Appendix 1.

### Analysis and Statistics

Drug monitoring parameters and tests were rated as concordant in discharge letters and the SmpC if (1) the drug monitoring parameter or test was stated explicitly in the SmPC and the discharge letter, for example, “measure potassium” or (2) if the SmPC or the discharge letter recommended drug monitoring parameters or tests that were related to each other. As an example, when the SmPC recommended potassium controls and the drug monitoring advices in the discharge letter recommended controls of electrolytes, these drug monitoring advices were also rated as concordant. The allocation of monitoring parameters was done independently by 2 investigators. The frequency of drug monitoring parameters and tests was determined and averages with SDs were calculated using Microsoft Excel. Cohen kappa was calculated to determine interrater reliability of the SIM score rating.

## Results

### Characteristics of the Included Discharge Letters and Discharge Medications

**Figure 1:**
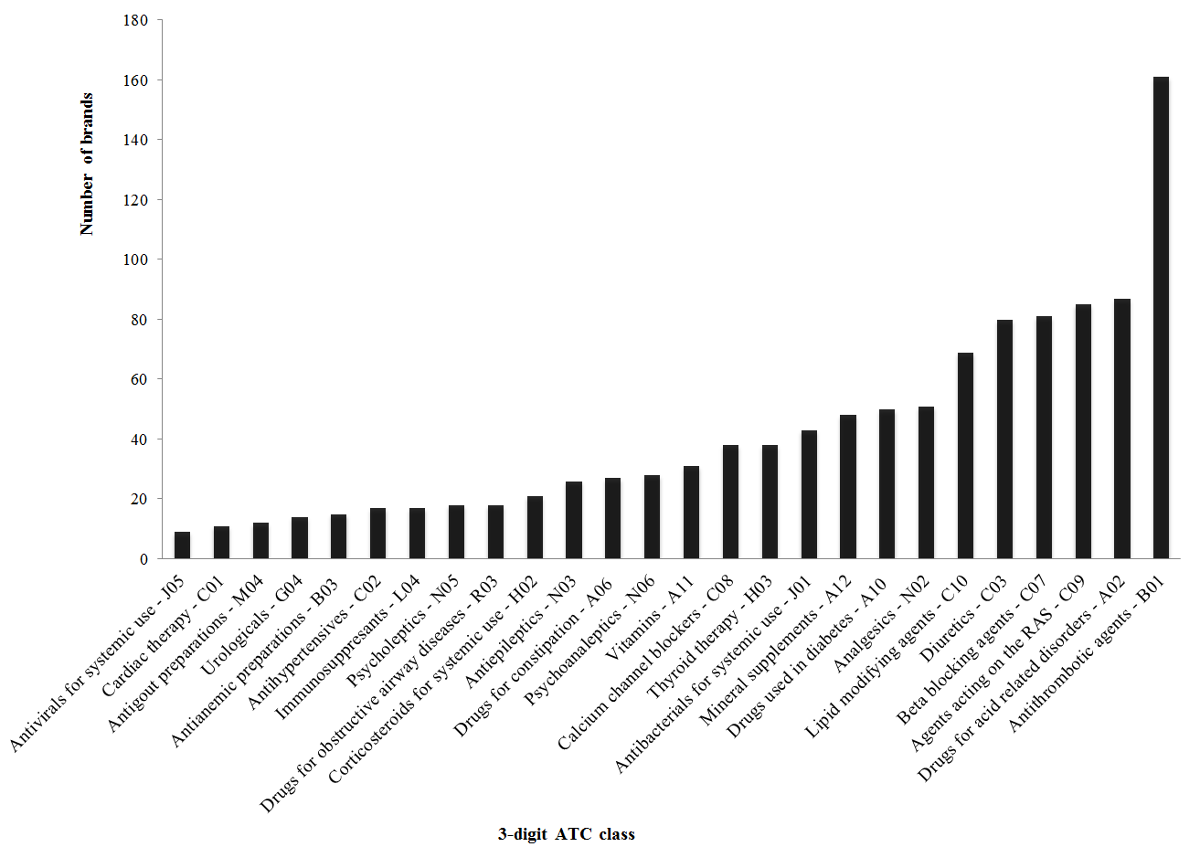
Most common prescribed drug groups (expressed as 3-digit anatomical therapeutic chemical code class) in 154 consecutive discharge letters of 3 large university departments (internal medicine, neurology, and surgery). ATC: anatomical therapeutic chemical code, RAS: renin-angiotensin system.

On the 2 index days, 158 discharge letters were issued and hence screened for inclusion. Yet, 4 of these discharge letters did not contain any discharge medication and were therefore excluded, leaving 154 discharge letters for analysis. There were 34 discharge letters from the surgery department (22.1%, 34/154), 95 from internal medicine (61.7%, 95/154), and 25 (16.2%, 25/154) from neurology. Overall, the discharge letters contained 1180 brands referring to 240 different active pharmaceutical substances from 51 different 3-digit anatomical therapeutic chemical code (ATC) groups (see Figure 1), resulting in an average of 7.7 (SD 4.3) brands per discharge letter. The most commonly prescribed brands were antithrombotic agents (B01, n=161), drugs for acid-related disorders (A02, n=87), agents acting on the renin-angiotensin system (C09, n=85), beta-blocking agents (C07, n=81), and diuretics (C03, n=80, Figure 1).

### Drug Monitoring Advices Provided in the Discharge Letter

Overall, 40 discharge letters (25.9%, 40/154) contained at least 1 drug monitoring advice for, in total, 57 brands (4.83%, 57/1180), and 29 active pharmaceutical substances (details are shown in Multimedia Appendix 2). Phenprocoumon (n=6), tacrolimus (n=5), and levothyroxine (n=5) were the active pharmaceutical substances most frequently accompanied by a drug monitoring advice (see Table 1). Drug monitoring advices most frequently suggested monitoring of renal function (n=9), trough concentrations (n=7), international normalized ratio (n=6), and blood glucose (n=6). Most drug monitoring advices were solely located in the text (n=34), some were included in the discharge medication (n=14), and the advices were rarely found in both text and discharge medication section (n=9; Table 1), leading to a total of 66 drug monitoring advices with a total of 69 suggested drug monitoring parameters and tests (referring to 18 different parameters and tests).

### Structure and Content of Drug Monitoring Advices in Discharge Letters

**Figure 2:**
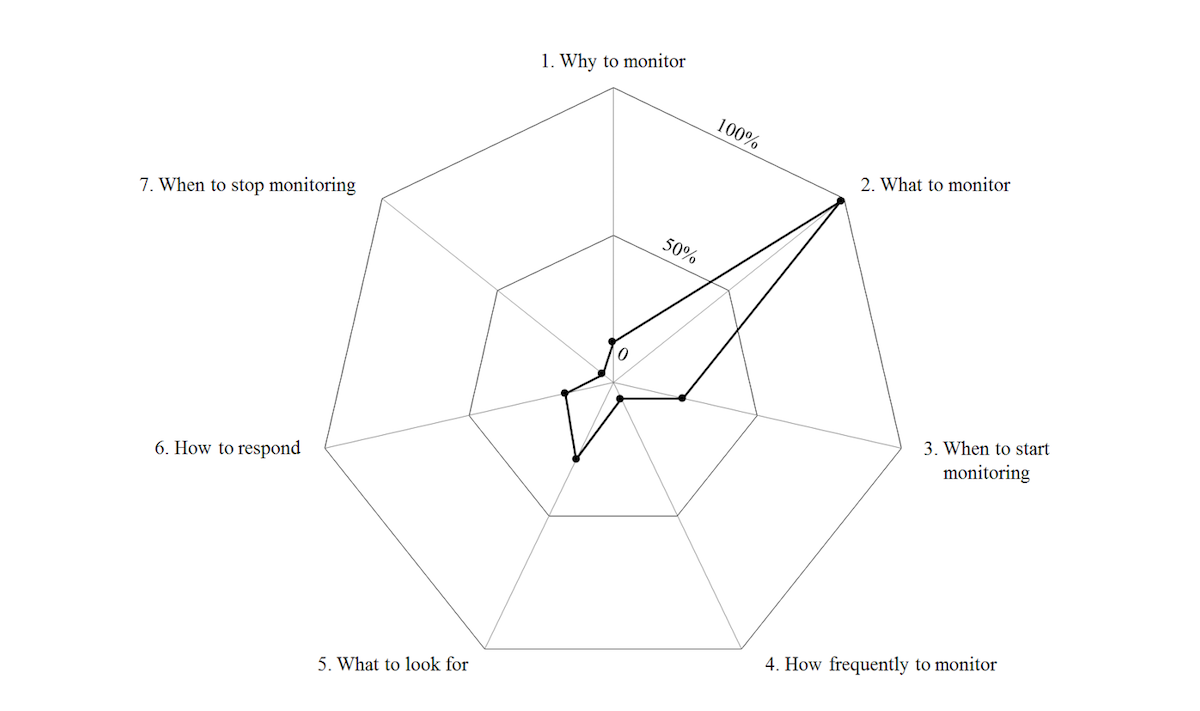
Overview of the frequency of systematic instructions for monitoring score domains used in the drug monitoring advices of the discharge letters.

Of the 66 drug monitoring advices, 20 addressed 1 domain, 29 addressed 2 domains, and 15 addressed 3 domains of the SIM score. Only 1 drug monitoring advice addressed 4 domains (what to monitor, when to start monitoring, how frequently to monitor, and when to stop monitoring), and 1 drug monitoring advice (ie, “we ask for regular endocrinological follow-up controls”) was too vague and hence did not meet any of the SIM domains. On average, the drug monitoring advices addressed 1.9 (SD 0.8) domains (see Figure 2).

Nearly all drug monitoring advices (99%, 65/66) contained a definition of the monitoring parameters or tests that should be performed. Around a quarter of the drug monitoring advices specified when drug monitoring should be started (24%, 16/66) and what should be looked for (29%, 19/66, Figure 2). Only few drug monitoring advices gave reasons of drug monitoring, that is, why to monitor (14%, 9/66), or described which actions to take in case of findings, that is, how to respond to deviations (17%, 11/66). Adequate timing of drug monitoring was seldom addressed; almost all drug monitoring advices lacked information on the frequency of monitoring, that is, how frequently to monitor (94%, 62/66) and when monitoring may be stopped (95%, 63/66). Interrater reliability was very good with a Cohen kappa of 0.89.

**Table 1:** Comparison of the drug monitoring parameters reported in the discharge letters with the monitoring recommendations in the corresponding summary of product characteristics.

Active pharmaceutical substance (frequency)^a^	Location in the discharge letter (frequency)^b^	Drug class	Drug monitoring parameter in discharge letter (frequency)^c^	Drug monitoring parameters in the summary of product characteristics
Apixaban (1/8)	TXT^d^ (1)	Factor Xa inhibitor	Blood cell count (1)	Bleeding signs
Atorvastatin (3/37)	MED^e^ (3), TXT (1)	HMG-CoA^f^-reductase inhibitor	CK^g^ (1), LDL^h^ (3), liver function (1)	CK, liver function test
Candesartan (1/20)	MED (1), TXT (1)	Angiotensin-II- receptor antagonist	Blood pressure (1), renal function (1)	Only in special patient populations (hypertension and impaired renal function, heart failure)
Carvedilol (1/10)	MED (1), TXT (1)	Nonselective beta blocker	Blood pressure (1), heart rate (1)	Only in special patient populations (heart failure with low blood pressure or ischemic heart disease)
Cefuroxime (1/4)	TXT (1)	Cephalosporin	Inflammatory parameters (1)	No parameters mentioned
Ciclosporin (2/2)	MED (1), TXT (1)	Calcineurin inhibitor	Blood concentration (2)	Serum potassium, serum magnesium, serum lipids, uric acid, renal function, liver function, ciclosporin concentrations, blood pressure, and physical examination
Ciprofloxacin (2/12)	TXT (2)	Fluorquinolone	Inflammatory parameters (2), renal function (1)	No parameters mentioned
Clindamycin (1/1)	TXT (1)	Lincosamide	Inflammatory parameters (1)	Blood cell count, liver function test, and renal function test
Colecalciferol (1/8)	TXT (1)	Vitamin	Serum calcium (1)	Calcium in serum and urine, creatinine
Dabigatran etexilate (1/3)	MED (1)	Thrombin inhibitor	Liver function (1), renal function (1)	Renal function, signs and symptoms of bleeding or anemia
Duloxetine (2/3)	TXT (2)	Selective serotonin and norepinephrine reuptake inhibitor	Serum sodium (2)	Only in special patient populations (old patients, hypertension, or heart disease)
Enoxaparin (2/36)	TXT (2)	Low-molecular-weight heparin	Blood cell count (2)	Platelet count
Eplerenone (1/4)	MED (1)	Mineralocorticoid receptor antagonist	Renal function (1), electrolytes (1)	Serum potassium
Furosemide (2/11)	TXT (2)	Loop diuretics	Renal function (1), electrolytes (1), body weight (1)	Potassium, sodium, calcium, bicarbonate, creatinine, blood urea, uric acid, and blood glucose
Hydrochlorothiazide (1/14)	MED (1), TXT (1)	Thiazide diuretic	Renal function (1), electrolytes (1),	Serum potassium, serum sodium, and serum magnesium
Insulin (6/30)	TXT (6)	Insulin	Blood glucose (6)	Blood glucose
Pancreatic enzyme supplement (2/6)	TXT(2)	Enzymes	Stool consistency (2)	No parameters mentioned
Levetiracetam (1/8)	TXT (1)	Antiepileptic	Renal function (1)	Suicidal ideation
Levothyroxine (5/34)	MED (1), TXT (5)	Thyroid hormone	Thyroid function (5)	No parameters mentioned
Nebivolol (1/7)	TXT (1)	Selective beta blocker	Heart rate (1)	No parameters mentioned
Oxcarbazepine (1/1)	TXT (1)	Antiepileptic	Serum sodium (1)	Serum sodium, suicidal ideation
Phenprocoumon (6/13)	MED (2), TXT (5)	Vitamin K antagonist	INR^i^ (6)	Liver function test, INR
Pravastatin (1/16)	MED (1)	HMG-CoA-reductase inhibitor	LDL (1)	Only in special patient population (patients with myopathy, impaired renal function, hypothyroidism, or alcohol abuse)
Ramipril (1/44)	MED (1)	Angiotensin-converting-enzyme inhibitor	Blood pressure (1)	Serum potassium, renal function, and leukocytes
Sildenafil (1/2)	MED (1)	Phosphodiesterase type 5 inhibitor	Blood pressure (1), heart rate (1)	No parameters mentioned
Simvastatin (1/9)	MED (1)	HMG-CoA-reductase inhibitor	LDL (1)	CK, liver function test
Spironolactone (2/13)	TXT (2)	Mineralocorticoid receptor antagonist	Renal function (1), electrolytes (1), body weight (1)	Potassium, sodium, calcium, bicarbonate, creatinine, blood urea, uric acid, and acid-base balance
Tacrolimus (5/6)	MED (5), TXT (2)	Calcineurin inhibitor	Blood concentration (5)	Electrolytes, liver function, renal function, fasting blood glucose, hematological parameters, coagulation, plasma proteins, blood concentration, blood pressure, ECG^j^, neurologic status, and vision
Torasemide (2/32)	MED (2), TXT (1)	Loop diuretic	Body weight (1), electrolytes (1), renal function (1)	Electrolytes, creatinine, uric acid, blood glucose, lipids, leukocytes, erythrocytes, and platelets

^a^The first number in parenthesis shows the number of active pharmaceutical substances with a drug monitoring advice; the second number in parenthesis indicates the total amount of discharge letters, which had the active pharmaceutical substance included.

^b^The number in parenthesis indicates how often the drug monitoring advice was located in the text or in the discharge medication.

^c^The number in parenthesis indicates how often the drug monitoring parameter was recommended for the corresponding active pharmaceutical substance.

^d^TXT: text.

^e^MED: discharge medication.

^f^HMG-CoA: hydroxymethylglutaryl-coenzyme A.

^g^CK: creatine kinase.

^h^LDL: low-density lipoprotein.

^i^INR: international normalized ratio.

^j^ECG: electrocardiogram.

### Comparison With Summary of Product Characteristics Information

For 52 of the 57 brands with an actual drug monitoring advice in the discharge letter, the SmPC also mentioned a drug monitoring advice, but of the 69 drug monitoring parameters and tests mentioned in the discharge letters, only 35 parameters (51%, 35/69) were also listed in the corresponding SmPC. In contrast, 29 of the 71 SmPC parameters (41%, 29/71) were included in the discharge letters, whereas the remaining 42 parameters (59%, 42/71) were not mentioned in the discharge letters at all (Table 1 and Multimedia Appendix 2). However, the SmPC suggested drug monitoring advices for many more drugs. Indeed, for 595 of the 1180 brands (50.42%) included in the discharge letters (referring to 132 out of 240 active pharmaceutical substances; 55.0% (132/240), the SmPC contained suggestions for drug monitoring that could be theoretically applied.

## Discussion

### Principal Findings

Drug monitoring advices were provided only for about 1 in 20 brands recommended in the discharge medication and most often did not offer all information domains a “best-practice” monitoring advice should contain. Following the SIM score, the advices only infrequently specified what to look for, why one should monitor, and what should be done in case of finding deviations. Regarding the timing of drug monitoring, a quarter of the advices specified a start of drug monitoring, but only 1 in 20 advices stated a frequency or defined an end of drug monitoring.

### Evidence Gap Regarding the Need of Drug Monitoring

To finally judge the quality of drug monitoring advices and also to subsequently derive an evidence-based information support tool, information on monitoring advices that have been shown to be clinically relevant is needed. Thereby, clinically relevant information might be particularly determined by the severity and probability of the potential ADE or efficacy loss as well as the chances that the ADE or efficacy loss can be reliably detected and prevented by the monitoring activity. The SmPC rather follows a generic approach and suggest up to 10 times more advices than were currently included in the letters. However, it remains unclear whether these advices are all clinically relevant and need to be followed in all patients [21,22]. Conversely, there are first hints suggesting that even the SmPC lacks relevant advices that are included in clinical guidelines. For instance, the 2016 heart failure guideline of the European Society of Cardiology recommends close monitoring of creatinine, serum potassium, and urea upon ACE inhibitor therapy initiation, which was not similarly mentioned in the German ramipril SmPC [23]. Although clinical guidelines might be expected to be a good source for clinically meaningful drug monitoring advices, this aspect is not a standard request for good guideline development, which mainly focuses on proper guideline development methods [24], and preliminary analyses suggest that drug monitoring advices are included only sporadically and certainly not systematically.

In daily practice, there are only few drugs with precise and unambiguous monitoring recommendations in the SmPC, for example, agranulocytosis monitoring with clozapine [25]. However, in most drugs, the monitoring need is vague and a specification requires clinical context factors such as (1) patient characteristics, (2) stage of therapy (eg, dose titration), and (3) comedication. This is also reflected by the discrepancies between the mentioned drug monitoring parameters and tests in the discharge letters and the SmPC in this study, which can be attributed to the evaluation of clinical context factors. As an example, blood glucose monitoring was recommended for a patient under insulin therapy in a discharge letter. This was consistent with the SmPC recommendations, but it also might appear rather obvious and lead to alert fatigue if integrated routinely in discharge letters. Regarding the clinical context factors, the respective patient had had pancreatectomy and therefore a clear clinical indication for close glucose monitoring in the postoperative phase, justifying the explicit drug monitoring advice.

This study therefore supports the hypothesis that there is an evidence gap in terms of a consistent definition of indications for drug monitoring and populations benefitting of it. Therefore, to close this gap, future research should address changes in ADE incidence over time and evaluate protective and risk factors that might have an impact on the need of drug monitoring. There are first approaches to develop such information tools, such as a recent recommendation providing suggestions for drug monitoring of high-risk medicines in primary care, which were derived from a range of guideline sources and expert opinions [20,26].

### Concept and Structure of Comprehensive and Practical Drug Monitoring Advices

If drug monitoring is indicated, the drug monitoring advices should be clearly formulated and support physicians in the development of individual monitoring plans. A comprehensive drug monitoring advice should follow the information clusters suggested in the SIM score [21]. The need of drug monitoring defined by a sole indication of a drug, for example, clozapine therapy, or a combination of clinical context factors define the domains “why to monitor” (SIM score domain 1) and what to monitor (SIM score domain 2). The stage of therapy (eg, drug initiation, maintenance, or tapering) is an important determinant regarding the proper timing of drug monitoring activities and specifies the start (SIM score domain 3), the frequency (SIM score domain 4), and the end of drug monitoring (SIM score domain 7). Timing is a crucial aspect of any monitoring because the risk of ADE varies over time as some drugs have a high risk of ADE early after drug initiation, for example, hyperkalemia with ramipril intake or dosage changes, whereas, other ADE more likely occur after longer time periods, for example, pulmonary toxicity caused by amiodarone [27-30]. The SIM score domain “what to look for” (SIM score domain 5) and “how to respond” (SIM score domain 6) are domains that were rarely addressed and, if addressed, sufficient information was lacking. For instance, the drug monitoring advice “please check liver function” lacks detailed information on what explicitly to look for because drug-induced liver injury occurs in different clinical patterns such as hepatic, cholestatic, or mixed, which can easily be detected by characteristic laboratory patterns [31,32].

### Limitations of the Study Design

This study has several limitations. First, a sample of 154 discharge letters was analyzed, which could be deemed as relatively small. To ensure representability, we included consecutive discharge letters of 2 working days rather than deliberately choosing letters of different patient populations; this approach covered a broad range of different brands (n=1180) and a sizeable number of different 3-digit ATC-codes (n=51). Moreover, the sample size was estimated on the basis of previous studies analyzing the quality and structure of drug monitoring advices in drug labels, which had similar or even lower sample sizes [21,22,33]. Second, direct clinical implications of missing drug monitoring parameters neither were nor could have been assessed in this study, and they neither were in the focus of our study. Furthermore, the clinical implications of infrequent drug monitoring are well known, and there is no obvious reason to omit proper monitoring of pharmacotherapy after discharge from tertiary care [3,34]. Therefore, the study focused on the structure and content of drug monitoring advices at interfaces of care to analyze potential areas for improvement and interventions, targeting the problem of infrequent drug monitoring in patient care. Third, we did not consider the date of onset of a specific drug as this information was scarcely available in the discharge letters. ADE of some active pharmaceutical substances (eg, hyperkalemia with ramipril intake) might occur more likely during dosage titration and monitoring periods could be longer, when long-term maintenance doses are taken uneventfully [27,29]. Consequently, it might be possible that the real monitoring need was overestimated or, on the other hand, that drug monitoring advices were not precise enough. Finally, we solely evaluated the drug monitoring advices of one other data source, that is, the SmPC. Yet, as the legally binding document also in terms of drug therapy monitoring, it could be the first reference consulted by prescribers and information therein should be reliable also in this regard.

### Conclusions

Drug monitoring advices were included in discharge letters only for a minority of brands; however, respective SmPC information was broad and unspecific in most parts, suggesting that a future monitoring database should consider not only the drug and its indication but also further patient characteristics, the stage of therapy, and the comedication.

## Appendix

Multimedia Appendix 1 Overview of summary of parameters, tests, and symptoms to monitor mentioned in the summary of product characteristics.

Multimedia Appendix 2 Overview of the drug monitoring advices recommended in 154 of 158 consecutive discharge letters of a university hospital.

## Abbreviations

ACE: angiotensin-converting-enzyme
ADE: adverse drug event
ATC: anatomical therapeutic chemical code
CK: creatine kinase
ECG: electrocardiogram
HMG-CoA: hydroxymethylglutaryl-coenzyme A
INR: international normalized ratio
LDL: low-density lipoprotein
MED: discharge medication
SIM: systematic instructions for monitoring
SmPC: summary of product characteristics
TXT: text
