# Supplementary material for: Structure and Content of Drug Monitoring Advices Included in Discharge Letters at Interfaces of Care: Exploratory Analysis Preceding Database Development
Source: JMIR Med Inform. 2019 Apr 8;7(2):e10832. doi: 10.2196/10832 (PMC6475819; doi:10.2196/10832)
Supplement: Multimedia Appendix 1 [file medinform_v7i2e10832_app1.pdf]

Title: Structure and content of drug monitoring advices included in discharge letters at interfaces of care – an exploratory analysis preceding database development

Journal name: JMIR Medical Informatics

Authors: B Morath [1, 2, 3], K Wien[1, 2], T Hoppe-Tichy [2, 3], W E Haefeli [1,2], H M Seidling [1,2]

[1]Department of Clinical Pharmacology and Pharmacoepidemiology, Heidelberg University, Im Neuenheimer Feld 410, 69120 Heidelberg, Germany

[2]Cooperation Unit Clinical Pharmacy, Heidelberg University, Im Neuenheimer Feld 410, 69120 Heidelberg, Germany

[3]Hospital Pharmacy, Heidelberg University Hospital, Im Neuenheimer Feld 670, 69120 Heidelberg, Germany

Corresponding author: Hanna Seidling | email: hanna.seidling@med.uni-heidelberg.de

Multimedia appendix I: Summary of parameters, tests, and symptoms to monitor mentioned in the summary of product characteristics

Table 1: Cluster of parameters, tests, and symptoms to monitor mentioned in the summary of product characteristics.

Superordinate category	Monitoring parameters in the SmPC
**Acid-Base-status**	
	pH (blood)
**Antibody**	
	Anti-nuclear antibody
	Smooth-muscle antigen antibody
**Brain**	
	Electroencephalography
**Body weight**	
	Body weight
**Carbohydrate metabolism**	
	Blood glucose
	Hemoglobin A1c
	Metabolic profile (Not further defined in SmPC)
**Coagulation**	
	Activated partial thromboplastin time
	International normalized ratio
	Partial thromboplastin time
	Platelet function
**Differential blood count**	
	Leucocytes
	Neutrophil granulocytes
**Electrolytes**	
	Bicarbonate
	Calcium
	Chloride
	Potassium
	Magnesium
	Sodium
	Phosphate
	Pyrophosphate
**Fluid balance**	
	No specific tests or parameters provided
**Heart function**	
	Blood pressure
	Electrocardiogram
	Heart rate
	Heart rhythm
**Inflammation parameters**	
	No specific tests or parameters provided
**Lipid parameter**	
	Cholesterol
	Triglycerides
**Liver function**	
	Alkaline phosphatase
	Bilirubin
	Transaminases
	Gamma-glutamyltransferase
	Alanine amino transferase
	Aspartate amino transferase
	Serum albumin
**Mineral metabolism**	
	Ferritin
	Parathormone
	Zinc

	Copper
	Iron status
**Muscular system**	
	Creatine kinase
**Pancreatic function**	
	Amylase
**Pulmonary function**	
	No specific tests or parameters provided
**Prostate**	
	Prostate-specific antigen
**Renal function**	
	Glomerular filtration rate
	Creatinine clearance
	Plasma cystatin C level
	Proteinuria
	Urea
	Blood urea
	Creatinine
**Small blood cell count**	
	Erythrocytes
	Leucocytes
	Platelets
	Hematocrit
	Hemoglobin
	Peripheral blood smear
**Symptoms and syndromes**	
	Agranulocytosis
	Anemia
	Atrial fibrillation
	Bleeding
	Clinical worsening of health status
	Diabetes
	Drug abuse
	Extrapyramidal disorders
	Fever
	Flu-like symptoms
	Hearing ability
	Hypersensitivity
	Hyperthyroidism
	Hypothyroidism
	Leucopenia
	Lymphadenopathy
	Skin reaction
	Sore throat
	Stomatitis
	Suicidal ideation
	Thrombocytopenia
	Vision
**Therapeutic drug monitoring**	
	Tacrolimus trough level
	Ciclosporin level
	Antiepileptic trough level
	Carbamazepine trough level
	Digitoxin trough level
	Digoxin trough level
	Methotrexate trough level
**Thyroid function**	
	Thyroid stimulating hormone

	Liothyronine
	Thyroxine
**Uric acid**	
	Uric acid
**Urinalysis**	
	Uric acid
	Calcium
	Glucose
	Urinary sediment
	Phosphate
	Pyrophosphate
	Specific weight
**Vitamins**	
	Vitamin A level
	Vitamin D level
	Vitamin E level
	Vitamin K level
	Vitamin B level

SmPC: summary of product characteristics
