# Supplementary material for: Structure and Content of Drug Monitoring Advices Included in Discharge Letters at Interfaces of Care: Exploratory Analysis Preceding Database Development
Source: JMIR Med Inform. 2019 Apr 8;7(2):e10832. doi: 10.2196/10832 (PMC6475819; doi:10.2196/10832)
Supplement: Multimedia Appendix 2 [file medinform_v7i2e10832_app2.pdf]

Title: Structure and content of drug monitoring advices included in discharge letters at interfaces of care – an exploratory analysis preceding database developement

Journal name: JMIR Medical Informatics

Authors: B Morath [1, 2, 3], K Wien[1, 2], T Hoppe-Tichy [2, 3], W E Haefeli [1,2], H M Seidling[1,2]

[1]Department of Clinical Pharmacology and Pharmacoepidemiology, Heidelberg University, Im Neuenheimer Feld 410, 69120 Heidelberg, Germany

[2]Cooperation Unit Clinical Pharmacy, Heidelberg University, Im Neuenheimer Feld 410, 69120 Heidelberg, Germany

[3]Hospital Pharmacy, Heidelberg University, Im Neuenheimer Feld 670, 69120 Heidelberg, Germany

Corresponding author: Hanna Seidling | email: hanna.seidling@med.uni-heidelberg.de

Supplementary II: Drug monitoring advices recommended in 154 of 158 consecutive discharge letters of a university hospital

Table 1: Detailed overview of all drug monitoring advices in the discharge letters

Active pharmaceutical substance	Location in the discharge letter	Text of the drug monitoring advice	SIMS Domains	Monitoring parameters SmPC
Apixaban 2.5 mg	TXT	There were two positive hemoccult tests under anticoagulation therapy. Please control blood count regularly.	2	Bleeding signs
Atorvastatin 10 mg *	TXT	Check CK, LDL, and the liver function within 4 weeks.	2	CK, liver function
Atorvastatin 10 mg*	MED	New, please check CK,LDL and liver function in 4 weeks.	3	CK, liver function
Atorvastatin 40 mg	MED	Target LDL < 70 mg/dl	1	
Atorvastatin 20 mg	MED	Target LDL < 70 mg/dl	1	
Candesartan 4 mg*	TXT	We recommend frequent blood pressure controls and the conduct of a 24 hour blood pressure test, if necessary. Please control blood pressure and renal function.	1	Only in special patient populations (hypertension and impaired renal function, heart failure)
Candesartan 4 mg*	MED	Please control blood pressure and renal function	1	
Carvedilol 3,125 mg*	TXT	We recommend frequent blood pressure controls and the conduction of a 24 hour blood pressure test, if necessary.	1	Only in special patient populations: Heart failure with low blood pressure or ischemic heart disease.
Carvedilol 3.125 mg*	MED	Please, control blood pressure and heart rate (target heart rate 60 beats per minute)	2	
Cefuroxime 500 mg	TXT	Please control inflammation parameters.	1	No monitoring parameters provided
Colecalciferol	TXT	We recommend close monitoring of serum calcium level.	1	Calcium in urine and serum, creatinine
Ciclosporin 75 mg	TXT	We recommend frequent controls of the blood concentrations and the adaption of the therapy, if necessary.	2	Ciclosporin concentrations, renal function, blood pressure, physical examination, serum lipids, serum potassium, serum magnesium, uric acid, liver function
Ciclosporin 125 mg	MED	Target blood concentration 80-100.	2	
Ciprofloxacin 250 mg	TXT	Please continue therapy until [date]. Subsequently, we recommend monitoring of inflammation parameters and renal function.	2	No parameters mentioned
Ciprofloxacin 500 mg	TXT	Please continue therapy until [date]. Subsequently, we recommend monitoring of inflammation parameters.	2	
Clindamycin	TXT	Please control inflammation parameters regularly.	1	Blood count, liver function, renal function
Dabigatran etexilate 110 mg	MED	New, lifelong. CAVE: Please control laboratory parameters, especially liver and renal function.	2	Renal function, signs and symptoms of bleeding or anemia
Duloxetine	TXT	Additionally, we recommend weekly controls of serum sodium concentrations (after each dosage increase) until the target dose is reached.	4	Only in special patient populations (old patients, hypertension or heart disease)
Duloxetine	TXT	Please control serum sodium concentrations weekly in primary care during the treatment course.	3	
Enoxaparin 40 mg	TXT	We recommend the continuation of the thrombosis prophylaxis e.g. enoxaparin 40 mg (0-0-1) for the next two weeks after discharge under regular control of blood cell count and clotting parameters.	3	Platelet count

Enoxaparin 40 mg	TXT	We recommend the continuation of the thrombosis prophylaxis e.g. enoxaparin 40 mg (0-0-1) for the next two weeks after discharge under regular controls of blood cell count and clotting parameters.	3	
Eplerenone 25 mg	MED	New, control electrolytes and renal function.	2	Serum potassium
Furosemide 40 mg	TXT	Please continue therapy with frequent monitoring of the electrolytes and the renal function.	2	Potassium, sodium, calcium, bicarbonate, creatinine, blood urea, uric acid and blood glucose
Furosemide 40 mg	TXT	We recommend the stepwise reduction of the diuretics under body weight control.	1	
Hydrochlorothiazide 12,5 mg *	TXT	Check renal function and electrolytes.	1	Serum potassium, serum magnesium
Hydrochlorothiazide 12.5 mg*	MED	Please control electrolytes and renal function.	1	Serum potassium, serum magnesium
Insulin	TXT	Because of the patient’s age, we recommend fasting blood glucose levels of 150 mg/dl. Please reevaluate during the course of treatment.	3	Blood glucose
Insulin	TXT	Because of the patient’s age, we recommend fasting blood glucose levels of 150 mg/dl. Please reevaluate during the course of treatment.	3	
Insulin	TXT	Close control of the blood glucose levels and an adaption of the dosage	2	
Insulin	TXT	We ask for close controls of the blood glucose levels and an adaption of the dosage within the everyday life.	2	
Insulin	TXT	Please adjust the blood glucose levels according to endocrinological recommendations.	1	
Insulin	TXT	Concerning the pancreoprive diabetes mellitus, we recommend diabetes training and the adjustment of the blood glucose levels according to endocrinological recommendations.	2	
Levetiracetam 500 mg	TXT	Renal function test at least twice yearly.	3	Suicidal thoughts
Levothyroxine 25 µg	TXT	Thyroid function should be checked.	1	No monitoring parameters provided
Levothyroxine 100 µg*	TXT	We ask for regular endocrinological follow-up controls	0	
Levothyroxine 75 µg	TXT	Because of the low T3 constellation under preexisting levothyroxine therapy, we ask for controls of TSH, T3 and T4 in 4 weeks.	3	
Levothyroxine 75 µg	TXT	Check TSH in 3-6 weeks after the reduction of the L-thyroxine dose.	3	
Levothyroxine 50 µg	TXT	Check thyroid function in 4 weeks and adjust dose, if necessary.	3	
Levothyroxine 100 µg*	MED	If necessary adjust according to TSH levels. Target TSH levels 0.1-0.2 mU/l	3	
Nebivolol 5 mg	TXT	We recommend the evaluation of the heart frequency profile with a 24 hour ECG and a titration of the beta blocker to a target resting heart rate of 60-70 beats per min, if necessary.	3	No monitoring parameters provided
Oxcarbazepine 300 mg	TXT	Regular controls of serum sodium levels.	1	Serum sodium, suicidal thoughts
Pancreatic enzymes	TXT	Dose adjustment depending on stool consistency	2	No monitoring parameters provided
Pancreatic enzymes	TXT	Dose adjustment depending on stool consistency	2	
Phenprocoumon 3mg	TXT	Target INR 2-3	2	Liver parameters, INR

Phenprocoumon 3mg*	TXT	Target INR of 2-3, the last INR was 3. Please adjust dose and check INR regularly.	3	
Phenprocoumon 3mg	TXT	Target INR 2-3	2	
Phenprocoumon 3mg	TXT	We recommend restarting the previous oral anticoagulation with phenprocoumon with a target INR of 2-3.	2	
Phenprocoumon 3mg	TXT	Please start oral anticoagulation with a target INR of 2.5-3.5	2	
Phenprocoumon 3 mg*	MED	Target INR 2.5-3	2	
Phenprocoumon 3 mg	MED	Target INR 2.5-3	2	
Pravastatin 40 mg	MED	Target LDL < 70 mg/dl	2	Only in special patient populations (Patients with myopathy, impaired renal function, hypothyrosis or alcohol abuse)
Ramipril 2.5 mg	MED	Target blood pressure < 140/90	2	Serum potassium, renal function, leukocytes
Sildenafil	MED	Please increase dosage depending on blood pressure and heart rate	2	No monitoring parameters provided
Spironolactone 100 mg	TXT	Check electrolytes and renal function.	1	Potassium, sodium, calcium, bicarbonate, creatinine, blood urea, uric acid, acid-base balance
Spironolactone 100 mg	TXT	Stepwise reduction of diuretics under body weight control.	1	
Simvastatin	MED	Target LDL < 100 mg/dl	2	CK, liver function test
Tacrolimus 1.5 mg*	TXT	Close control of blood concentrations and dose adjustment accordingly, if necessary. Please check tacrolimus trough concentrations in 3-4 days.	3	Blood concentration, blood pressure, ECG, neurologic status, visual faculty, fasting blood glucose, electrolytes, liver function, renal function, hematological parameters, blood clotting, plasma proteins
Tacrolimus 1 mg*	TXT	Laboratory assessment of inflammation parameters, blood cell count, and blood concentration of tacrolimus on Wednesday [date].	3	
Tacrolimus 1 mg	MED	Target trough concentration 3-5 ng/ml	2	
Tacrolimus 1 mg	MED	Target trough concentration 3-5 ng/ml	2	
Tacrolimus 1 mg	MED	Control trough concentration	1	
Tacrolimus 1.5 mg*	MED	Target trough concentration 10-15 ng/ml	2	
Tacrolimus 1 mg*	MED	Please control trough concentration in 3-4 days	2	
Torasemide 10mg*	TXT	Please check electrolytes and renal function.	1	electrolytes, creatinine, uric acid, blood glucose, lipids, leukocytes, erythrocytes and thrombocytes
Torasemide 10 mg*	MED	Please control electrolytes and renal function.	1	
Torasemide 10 mg	MED	Daily body weight control.	1	

Abbreviations: ECG: Electrocardiogram; **INR**: international normalized ratio; **LDL:** Low density lipoprotein; **MED:** Discharge medication section of the discharge letter; **SIMS**: SIM-Score **TXT:** Text section of the discharge letter; **T3:** Triiodothryronine; **T4:** Thyroxine; **TSH:** Thyroid stimulating hormone.
Legend: *Drug monitoring advice was provided for the brand in both the discharge medication and the text of the discharge letter.
